# Analysis of residents’ food safety satisfaction from the perspective of income heterogeneity

**DOI:** 10.1038/s41598-021-85384-2

**Published:** 2021-03-23

**Authors:** Xianmei Li, Xiandong Li, Yuanlin Liao, Guanghui Zhu, Guoxin Yu

**Affiliations:** 1 School of Economics and Trade, Xinjiang Agricultural University, Ürümqi, 830052 Xinjiang China; 2 Xinjiang Uygur Autonomous Region Strategic Research Institute, Ürümqi, 830011 Xinjiang China; 3 Postdoctoral mobile station of agricultural and forestry economic management, Xinjiang Agricultural University, Ürümqi, 830052 Xinjiang China

**Keywords:** Environmental social sciences, Health care

## Abstract

Food is the paramount necessity of the people, while food safety is the priority. Facing the increasingly serious food safety problems in China, how to improve food safety has become the responsibility of the whole society, and also the obligation of social development. Based on the 8000 residents’ survey data, the ordinal logistic model was used to analyze the residents’ satisfaction with food safety. The results show that residents are generally worried about food safety, and there is a strong demand for safe food. Gender, age, educational background, supervision, publicity, and complaint handling significantly affect residents’ food safety satisfaction. In terms of income heterogeneity, low-income residents have the highest degree of satisfaction with food safety. With the improvement of income level, their degree of satisfaction is decreasing. The high-income class residents have a strong consciousness of rights safeguarding after purchasing unsafe food. For low-income residents, their awareness of rights protection is declining due to insufficient income. In the way of rights protection, most respondents choose to return or refund money after negotiation with the seller. The lack of punishment for unscrupulous sellers is one of the main reasons for the frequent occurrence of food safety problems in China.

## Instruction

Food consumption is the most fundamental consumption item in resident life. Eating safe and healthy food is not only the basic right of each citizen, but also the basic obligation of national departments of food supervision, the enterprises of food production, processing and circulation. Food safety loopholes have exploded in China for the past few years. As a focus of social concerns, they have brought serious harms to residents’ health^1^.

The Food Safety Law of the People's Republic of China was adopted at the 7th Meeting of the Standing Committee of the National People's Congress in 2009, which was implemented on June 1. By 2015, this law had been in force for 6 years. In the meantime, a lot of scholars conducted detailed researches on governmental supervision, legal system, and standard system of food safety. For example, Andrew analyzed how to build socioeconomic gradients of health satisfaction from the perspective of heterogeneity^2^. Based on the heterogeneity of nationalities and religions, Oguzhan explored such issues as the equality of policy enforcement and the redistribution of social resources^3^. Erdem studied the impacts of heterogeneity on the voluntary dedication and welfare of dynamic public interest^4^. Francisca Antman studied the characteristics of heterogeneous poverty trap and nonlinear incomes from the dynamic perspective^5^. Mette used panel data to analyze the heterogeneity of the demand and income effect of Spanish consumers^6^. In the study of food safety, domestic scholars including Liang et al. studied the satisfactions of resident food safety with the cases of residents in Hubei Province, Taiyuan, etc. Thereafter, the researches on satisfactions of food safety highlighted the combination of metering methods and empirical analysis^7^. Such Wang, Wei et al. respectively applied such methods as ordinal logistic regression analysis and dichotomy logistics regression analysis to conduct quantitative research on consumer satisfaction with food safety^8,9^. Glynn analyzed the consumers’ satisfaction with beef safety in such countries as Canada, Japan, Mexico, and USA^10^. The researches of other scholars focused on the promotion of food safety measures. Trepka held the opinion that we could promote the satisfaction of consumers with food safety by setting up interactive multi-media information pavilions and timely solving the food safety problems of consumers^11^. John held the opinion that the FDA Food Safety Modernization Act (FSMA)reduced the risks of agricultural products to human health from the breakout of foodborne diseases by bringing in compulsory measures of agricultural prevention and control^12^. From the perspective of food consumer chain, Sara held the opinion that we could effectively control food quality and promote consumers’ satisfactions with food safety by supervising devices of food production, processing and transportation, the personal hygiene of employees, food treatment, and other systems, offering technical training to staff and carrying out regular and irregular examination^13^. Basem held the opinion that the quality and safety of food can be ensured by setting up a strict system of food safety traceability, reinforcing the traceability and punishment over inconformity with food safety standards, and forming the self-restraint of the industry^14^.

According to the literature reviews, the Law of Food Safety of China has been in force for a relatively short time, with lagging impacts on residents. It is imperative to make due investigation and analysis to the status of food safety satisfaction of consumers in recent years. Next, the heterogeneity of residents’ income is not distinguished in the study of residents’ (consumers') food safety satisfaction. Therefore, according to residents’ income heterogeneity, it is divided into three stratums: I. CNY 0–30,000, the low-income stratum; II. CNY 30,001–70,000, the medium-income stratum; III. Over CNY 70,000, the high-income stratum, to study the residents’ satisfaction with food safety and its influencing factors.

## Materials and methods

### Data source and sample distribution

The data used in this paper is provided by Professor Yu Guoxin of Xinjiang Agricultural University, who commissioned the Statistics Information Reference Center (SIRC) of the subsidiary of Xinjiang Uygur Autonomous Region Food and Drug Administration. According to our research needs, they take the random sampling survey by GW-CATI system (Computer Aided Telephone Interview System) and obtain the survey data. At the same time, we are very grateful for technical and data support provided by SIRC.

CATI is a system composed of computer network, hardware system and survey software. Through the computer network and the telephone connection, random dialing is realized; through the survey software, it can be realized on the computer to generate survey questionnaires, control sample composition, complete data summary analysis, and monitor the visit process. The computer-assisted telephone survey system can help us overcome the limitations of geographic space and increase sample coverage. Even residents living in remote mountainous areas, we can interview them by telephone. According to our research objectives, we selected permanent residents who have lived in the local area for more than 6 months and aged between 16 and 65 as the survey samples. Our sample covers 14 prefecture-level cities and 5 municipalities (Shihezi, Alar, Tumushuke, Wujiaqu, and Beitun) of Xinjiang Autonomous Region.

The investigation items include: the appraisal of consumers to the situation of food safety, the appraisal of consumers to the supervision of food safety, the consciousness and behaviors of consumers for food safety, and the right-safeguarding means and other basic information of consumers. In order to ensure the quality of the data, we adopted preliminary and re-examination to review all survey questionnaires. The error rate of the questionnaire was not higher than 2%. The samples with serious lack of information and unclear question answers were re-interviewed and improved. After the questionnaire review, all questionnaires will be randomly checked at a rate of 15% to improve the quality of the sample. A total of 8,000 valid samples has obtained in this study. Among them, Urumqi has the largest sample size, reaching 1,400, accounting for 17.5% of the total sample size, followed by Kashgar City (861), Ili Prefecture (816), Hotan City (765), Aksu City (676), and Changji Prefecture (519). The sample size of the survey in the eight regions of other provinces is less than 500. For the five municipalities directly under the Central Government, we obtained 100 valid samples, accounting for 6.3% of the total samples (See Table 1).

**Table 1:** The number of samples and their distribution.

Region	Sample size	Proportion (%)	Region	Sample size	Proportion (%)
Shihezi City	190	2.4	Bortala Mongol Autonomous Prefecture	165	2.1
Hami Prefecture	285	3.6	Bayingolin Mongol Autonomous Prefecture	485	6.1
Ili Prefecture	816	10.2	Akesu Prefecture	676	8.5
Tacheng Prefecture	455	5.7	Kizilsu Kirghiz Autonomous Prefecture	220	2.8
Altay Prefecture	278	3.5	Hui Autonomous Prefecture of Changji	519	6.5
Kashi Prefecture	861	10.8	Tumshuq City	100	1.3
Hotan Prefecture	765	9.6	Urumqi City	1400	17.5
Wujiaqu City	100	1.3	Karamay City	195	2.4
Alear City	100	1.3	Turpan Prefecture	290	3.6
Beitun City	100	1.3			

Data source: The Statistics Information Reference Center of Xinjiang (SIRC).

### Sample overview

In terms of gender structure, there are more female samples than male samples. The percentage of male samples is 42.1% which is lower 15.8% than female samples. Because we choose the target population as a regular purchase of food in the population, this work in China is completed by the family of women, so the proportion of women in the questionnaire is higher. In terms of age structure, the percentage of the samples aged 31–45 is the highest was reaching 39.7%. The sample aged 46–65 had the lowest proportion was 28.1%. In terms of educational background structure, more than 75.8% of samples have a high school education or above, and it has a relatively strong understanding of the questionnaire content, which is beneficial for reflecting the actuality of residents. In terms of occupational distribution, the occupations of interviewed residents cover a wide scope, with sample percentages fluctuating around 20%. In terms of the occupations of respondents, the survey data can comprehensively reflect the satisfaction of residents of different occupations with food safety. In terms of income heterogeneity characteristics, the low-income group accounted for the highest proportion which is 41.2%. The percentage of medium-income stratum accounts for 38.3%. The percentage of high-income stratum is 20.5% (See Table 2).

**Table 2:** Basic information about respondents.

Item	Percentage (%)	Item	Percentage(%)
**Sex**		**Educational background**	
1 = Male	42.1	1 = Senior college and above	25.30
2 = Female	57.9	2 = Junior college	28.80
**Age(years old)**		3 = Senior high school	21.70
1 = 16–30	32.2	4 = Junior high school and below	24.20
2 = 31–45	39.7	**Occupations**	
3 = 46–65	28.1	1 = Employees of enterprises and institutions	42.70
**Annual family income(Y,10,000 yuan)**		2 = Technicians	2.90
1 = 0 ≤ Y ≤ 3	41.2	3 = Service workers	16.80
2 = 3 < Y ≤ 7	38.3	4 = Rural migrant workers in cities	24.20
3 = Y > 7	20.5	5 = Other people	13.40

Data source: SIRC.

## Research methods

The paper introduces the current situation of income heterogeneity food safety problems by using data descriptive statistical analysis. As well, the Ordered Logistic Model (*OLM*) was used to analyze the Factors to influence residents’ satisfaction with food safety^15^. If the response variable Y is ordinal, the categories can be ordered in a natural way, such as there are many contexts in which a variable is ordinal and there are three or more categories. Some typical examples are health status (very good, good, so-so, bad, very bad), political ideology (very liberal, slightly liberal, moderate, slightly conservative, very conservative), fertility intention (the more the better, two, one, no). Say Y is an ordinal dependent variable with c categories. Let Pr (Y ≤ i) denote the probability that the response on Y falls in category j or below (i.e., in category 1,2, …, or i). This is called a cumulative probability. It equals the sum of the probabilities in category j and below (1):

1 $${\text{P(Y }} \le {\text{ i) = P(Y = 1) + P(Y = 2 ) + }} \cdots {\text{ + P(Y = i )}}$$

A “c category Y dependent variable” has c cumulative probability: Pr(Y ≤ 1), Pr(Y ≤ 2), … Pr (Y ≤ i). The final cumulative probability adopts a full scale; as a consequence, Pr (Y ≤ i) = 1. The forming order of the final cumulative probabilities reflects the rank of the dependent variable scale, and those are satisfiability probability with itself: Pr (Y ≤ 1) ≤ Pr(Y ≤ 2) ≤ … ≤ Pr(Y ≤ i) = 1.

In ordered logistic, the potential probability fraction observed for the ith response category is estimated as a linear function of the independent variable and a set of threshold points (also called cut points)^16^. The probability of observing response category i corresponds to the probability that the estimated linear function, plus random error is within the range of the threshold points estimated for that response^17^. Multiple logistic regression models can be applied without using information about ordering. One way to take account of the ordering is the use of cumulative probabilities, cumulative odds, and cumulative logistic. Considering k + 1 ordered categories, these quantities are defined by Eqs. (2) and (3):

2 $$o{\text{dds(Y}} \le {\text{i) = }}\frac{{{\text{P(Y}} \le {\text{i)}}}}{{{\text{1 - P(Y}} \le {\text{i)}}}}{ = }\frac{{{\text{p}}_{{1}} + \cdots + p_{1} }}{{p_{i + 1} + \cdots + p_{k + 1} }}$$

3 $${\text{lgoit(Y}} \le {\text{i) = ln}}\left( {\frac{{{\text{P(Y}} \le {\text{i)}}}}{{{\text{1 - P(Y}} \le {\text{i)}}}}} \right){ ,}\quad {\text{ i}} = {1,} \ldots {\text{,k}}$$

The cumulative logistic model of ordinal response data is shown in Eq. (4).

4 $${\text{lgoit(Y }} \le {\text{ i) = }}\phi { + }\beta_{1} {\text{X}}_{{1}} { + }\beta_{2} {\text{X}}_{{2}} { + } \cdots { + }\beta_{{\text{m}}} {\text{X}}_{m} { ,}\quad {\text{i}} = {1,} \ldots {\text{,k}}$$

According to the relevant research results of McCullagh and Bender R, etc.^18,19^, the ordinal Logistic model was used to analyze the factors to influence the income heterogeneity of residents’ food safety satisfaction. In this paper, Eq. (5) was constructed as follows:

5 $$\begin{aligned} {\text{lgoit(Y)}} & { = }\beta_{1} {\text{X}}_{{1}} { + }\beta_{2} {\text{X}}_{{2}} { + }\beta_{3} {\text{X}}_{{3}} { + }\beta_{4} {\text{X}}_{{4}} { + }\beta_{5} {\text{X}}_{{5}} { + }\beta_{6} {\text{X}}_{{6}} { + }\beta_{7} {\text{X}}_{{7}} \\ & \quad { + }\beta_{8} {\text{X}}_{{8}} { + }\beta_{9} {\text{X}}_{{9}} { + }\beta_{10} {\text{X}}_{{{10}}} + \, \beta_{11} {\text{X}}_{{{11}}} { + }\beta_{12} {\text{X}}_{{{12}}} { + }\theta \\ \end{aligned}$$

where Y represents the residents’ food safety satisfaction evaluation level (satisfaction), β representative coefficient of estimated model parameter. X_1_ is used to express the residents’ evaluation of government supervision work on food safety. X_2_ represents the residents’ evaluation of government publicity work on food safety. X_3_ represents the residents’ evaluation of government efforts to combat food insecurity. X_4_ represents the residents’ evaluation of government special rectification work of food safety. X_5_ represents the residents’ comments on the government’s handling of consumer complaints. X_6_ represents the residents’ the level of concern about food safety. X_7_ represents the residents’ income level. X_8_ is the gender of the residents. X_9_ is the age of residents. X_10_ represents residents living in rural areas or towns. X_11_ represents the occupation of residents. X_12_ represents the education degree of residents. θ represents the random error term of model estimation.

Also, to further explore the residents' satisfaction with the food safety factors. The income level of residents in X7 is not included in Eq. (5) which includes low-income, middle-income class, and high-income residents, and to analyze the influencing factors by using the following Eq. (6). But the meaning of β_i_ and X_i_ has no change.

6 $$\begin{aligned} {\text{lgoit(Y)}} & { = }\beta_{1} {\text{X}}_{{1}} { + }\beta_{2} {\text{X}}_{{2}} { + }\beta_{3} {\text{X}}_{{3}} { + }\beta_{4} {\text{X}}_{{4}} { + }\beta_{5} {\text{X}}_{{5}} { + }\beta_{6} {\text{X}}_{{6}} \\ & \quad { + }\beta_{8} {\text{X}}_{{8}} { + }\beta_{9} {\text{X}}_{{9}} { + }\beta_{10} {\text{X}}_{{{10}}} + \, \beta_{11} {\text{X}}_{{{11}}} { + }\beta_{12} {\text{X}}_{{{12}}} { + }\theta \\ \end{aligned}$$

## Result and analysis

### The trust in the safety of basic food

With regard to the trust of residents in basic food consumption (See Table 3), in terms of food variety, rice and flour have the highest degree of reassurance, followed by vegetable, fruits, and bean products. The food around campus and the milk powder of infant formula have the lowest degree of reassurance. Residents show quite different degrees of trust in the same food variety. The results show that the trust of residents in rice and flour is 64.6%, and with concerns about its quality accounting for 11.3%. The degrees of trust of residents in vegetables, fruits, and bean products are relatively similar which being 54.1% and 53.6% respectively. The proportion of worry about the quality of vegetables and fruits is 20.5%, and the percentage of worry about the quality of bean products is 17.1%. The residents’ trust in aquatic products is 45.6%, and their concerns about the quality of aquatic products is 24.3%. The residents’ trust in edible oil is 44.9%, and the concerns about the quality of edible oil is 32.0%. The residents’ trust in meat products is 42.6%, and the worry about the quality of meat products is 32.3%. residents’ trust in food and beverage service was 29.1%and the concerns about the quality of catering service is 34.3%. The trust in milk powder of infant formula is 27.1%, and the proportion of worry about the quality of milk powder of infant formula is 44.7%. The lowest satisfaction of residents with child food around campus is 12.7%, with more than 75% of residents holding the opinion that they worry about the quality of the food around campus.

**Table 3:** Degrees of trust of residents in food safety Unit: %.

Food variety	Quite trustful	Relatively trustful	Average	Worried
Rice and flour	45.0	19.6	24.1	11.3
Vegetable and fruits	39.0	15.1	25.4	20.5
Bean products	38.3	15.3	29.3	17.1
Aquatic products	32.7	12.9	30.1	24.3
Edible oil	31.6	13.3	23.1	32
Meat products	30.4	12.2	25.1	32.3
Barreled drinking water	31.3	10.8	24.8	33.1
Alcoholic drinks	22.9	13.9	35.1	28.1
Catering service	19.2	9.9	36.6	34.3
Milk powder of infant formula	18.7	8.4	28.2	44.7
The food around campus	9.2	3.5	11.5	75.8

### Attention to food safety

In terms of attention of residents to food safety (See Table 4), the attentions of residents with heterogeneous incomes to food safety are all above 80%; therein, the attention of low-income stratum is 80.8%, the attention of medium-income stratum is 82.8%, and the attention of high-income stratum is 84.2%. In terms of samples of non-attention, 7.4% of low-income stratum residents pay no attention to food safety and the percentages of disinterest among medium-income and high-income stratum are 4.2% and 3.6% respectively. That is to say: the level of income heterogeneity exerts a significant impact on the level of residents’ concerns about food safety, and shows a trend of positive correlation. According to actual survey analysis, this is because food safety is directly related to residents’ health. As a carrier for engaging in production labor, the human body exerts a huge influence on daily production and life. Therefore, residents have relatively high attention to food safety.

**Table 4:** Attentions of residents to food safety Unit: %.

	Close attention	Enough attention	Average	No attention
Low-income stratum	64.7	16.1	11.8	7.4
Medium-income stratum	60.5	22.3	13.0	4.2
High-income stratum	58.2	26.0	12.2	3.6

### Right-safeguarding means

According to the investigation, it indicates that more than half of residents have suffered from unsafe food, with the percentage of low-income, medium-income, and high-income stratum being 52.7%, 63.3%, and 74.5% respectively. As analyzed from the right-safeguarding means applied by residents with heterogeneous incomes when suffering from infringement (See Table 5), the most common means of right-safeguarding are negotiating with operators and complaining to consumer associations, with the percentages of low-income, medium-income, and high-income stratum being 39.6%/14.3%, 47.4%/12.4%, and 49.4%/9.6% respectively. Residents with heterogeneous incomes generally choose to negotiate with operators to protect their rights, but cannot effectively solve their problems in this way. On the contrary, the methods which can effectively safeguard rights such as filing a lawsuit to the court or complaining to consumer associations, the percentage of selection is universally low and it is 0.3%, 0.3%, and 0.2% for low-income, medium-income, and high-income stratum respectively. The statistical data shows that the heterogeneous income residents who do not take action to protect their right also take a high proportion among low-income, medium-income, and high-income stratum, which accounting for 34.6%, 28.5%, and 30.8% respectively, which is one of the main reasons of growing food safety problems in China, meanwhile, it’s also related to the cost of filing a lawsuit to the court and other right-safeguarding means. Safeguarding rights by filing a lawsuit to the court is a waste of time also takes up a lot of energy and money. Therefore, ordinary consumers will not choose this means when suffering from a minor food safety issue. On the whole, residents with heterogeneous incomes perform quite differently in the selection of right-safeguarding means.

**Table 5:** The selection of right-safeguarding means Unit: %.

	Negotiating with operators	Complaining to consumer associations	Disclosing facts to the media	Filing a lawsuit to the court	Failing to take any right-safeguarding action
Low-income stratum	39.6	14.3	11.2	0.3	34.6
Medium-income stratum	47.4	12.4	11.4	0.3	28.5
High-income stratum	49.4	9.6	10.0	0.2	30.8

### Overall satisfaction with food safety

From the analysis of the overall satisfaction degree of residents with income heterogeneity on food safety (Table 6), the satisfaction rates of low-income, middle-income, and high-income residents is 53.0%, 42.1%, and 38.2%, respectively, and the dissatisfaction rate is 12.9. %, 14.6%, 15.4%. That is, the residents’ income level and satisfaction show a negative correlation. According to actual investigation and analysis, this phenomenon is related to the safety of the food itself. Another reason is that as the purchasing power of the high-income class increases, the demand for food quality and safety increases, which is the main reason for the low satisfaction of high-income residents.

**Table 6:** Overall satisfaction of residents to food safety Unit: %.

	Quite satisfied	Relatively satisfied	Average	Unsatisfied
Low-income stratum	31.5	21.5	34.1	12.9
Medium-income stratum	20.3	21.8	43.4	14.6
High-income stratum	15.8	22.4	46.4	15.4

## Analysis of influencing factors of satisfaction

SPSS19.0 software was used to establish an Ordered Logit model (5) to analyze the factors affecting residents’ satisfaction with food safety. Then, Model (6) was built to analyze the factors that influence the food safety satisfaction of residents with heterogeneous incomes. To select the input and expression method of a linear regression variable, select "Backward: Wald". First, all adjoint variables shall be added to the model. Then, the minimum variable was eliminated according to the statistical probability value of Wald, and the equation was re-fitted until all the impacts of the remaining concomitant variables on dependent variables pass the significance test. The parameter estimation results of the model (5) are shown in Table 7. When Model (6) is built, the impact of the occupation variable is not significant; therefore, Model (7) is rebuilt. The parameter estimation results are shown in Table 8.

7 $$\begin{aligned} {\text{lgoit(Y)}} & { = }\beta_{1} {\text{X}}_{{1}} { + }\beta_{2} {\text{X}}_{{2}} { + }\beta_{3} {\text{X}}_{{3}} { + }\beta_{4} {\text{X}}_{{4}} { + }\beta_{5} {\text{X}}_{{5}} { + }\beta_{6} {\text{X}}_{{6}} \\ & \quad { + }\beta_{8} {\text{X}}_{{8}} { + }\beta_{9} {\text{X}}_{{9}} { + }\beta_{10} {\text{X}}_{{{10}}} { + }\beta_{12} {\text{X}}_{{{12}}} { + }\theta \\ \end{aligned}$$

**Table 7:** Model (5) parameter estimation.

	βi	Std. error	Wald	EXP (βi)	95%confidence limits	
					Lower limit	Upper limit
**Threshold**						
Y = 1 Quite satisfied	2.523***	0.129	383.323	–	2.271	2.776
= 2 Relatively satisfied	4.092***	0.135	919.352	–	3.828	4.357
= 3 Average (= other is control group)	6.973***	0.149	2199.411	–	6.682	7.265
**Location**						
X_1_ Supervision	1.146***	0.031	1408.621	3.15	1.086	1.206
X_2_ publicity	0.112***	0.032	11.961	1.12	0.049	0.176
X_3_ punishment intensity	0.334***	0.033	105.204	1.40	0.27	0.397
X_4_ Special rectification	− 0.068**	0.032	4.37	0.93	− 0.131	− 0.004
X_5_ complaint settlement	0.188***	0.024	63.224	1.21	0.142	0.235
X_6_ Attention	− 0.028	0.025	1.275	0.97	− 0.078	0.021
X_7_ Income level	0.059**	0.027	4.715	1.06	0.006	0.112
X_8_ Gender 1 = Male (female is control group)	− 0.249***	0.045	30.411	0.78	− 0.338	− 0.161
X_9_ Age 1 = 16–30	− 0.403***	0.062	41.842	0.67	− 0.525	− 0.281
2 = 31–45 (other is control group)	− 0.111**	0.056	3.947	0.89	− 0.221	− 0.001
X_10_ Residential area 1 = urban (other is control group)	0.157***	0.062	6.301	1.17	0.034	0.279
X_11_ Occupation1 = enterprises and employees	− 0.156**	0.073	4.533	0.86	− 0.299	− 0.012
2 = Technicians	− 0.225	0.144	2.431	0.80	− 0.507	0.058
3 = Rural migrant workers in cities	− 0.262***	0.085	9.42	0.77	− 0.429	− 0.095
4 = Service workers (other is control group)	− 0.108	0.075	2.085	0.90	− 0.255	0.039
X_12_ Education 1 = Senior college and above	0.322***	0.077	17.67	1.38	0.172	0.473
2 = Junior college	0.213***	0.068	9.742	1.24	0.079	0.347
3 = Senior high school (other is control group)	0.201***	0.069	8.605	1.22	0.067	0.335
Only intercept finally	-2 Log likelihood value		Chi-square		Pseudo R-squared	
	20,517.431		–		Cox & Snell	0.432
	15,989.39		4528.041***		Nagelkerke	0.466
					McFadden	0.215

Note: ***P < 0.01, **P < 0.05, *P < 0.1, the same below.

Link function: Logit.

**Table 8:** Model (7) parameter estimates.

Variables	Low-income stratum			Medium-income stratum			High-income stratum		
	βi	Std. Error	EXP (βi)	βi	Std. Error	EXP (βi)	βi	Std. Error	EXP (βi)
**Threshold**									
Y = 1 Quite satisfied	2.496***	0.181	–	2.274***	0.274	–	2.351***	0.247	–
= 2 Relatively satisfied	3.884***	0.19	–	3.926***	0.286	–	4.166***	0.258	–
= 3 Average (= other is control group)	6.47***	0.211	–	6.961***	0.315	–	7.303***	0.281	–
**Location**									
X1 Supervision	1.029***	0.044	2.80	1.078***	0.063	2.94	1.369***	0.057	3.93
X2 publicity	0.124***	0.048	1.13	0.171***	0.068	1.19	0.047	0.059	1.05
X3 punishment intensity	0.3***	0.048	1.35	0.354***	0.067	1.42	0.377***	0.06	1.46
X_4_ Special rectification	− 0.056	0.05	0.95	0.009	0.067	1.01	− 0.13**	0.056	0.88
X_5_ complaint settlement	0.213***	0.036	1.24	0.222***	0.05	1.25	0.129***	0.04	1.14
X_6_ Attention	− 0.03	0.037	0.97	0.014	0.054	1.01	− 0.08*	0.046	0.92
X_8_ Gender 1 = Male (female is control group)	− 0.168**	0.072	0.85	− 0.31***	0.093	0.73	− 0.31***	0.075	0.73
X_9_ Age 1 = 16–30	− 0.52***	0.096	0.59	− 0.43***	0.131	0.65	− 0.194*	0.109	0.82
2 = 31–45 (other is control group)	− 0.099	0.085	0.91	− 0.098	0.117	0.91	− 0.074	0.098	0.93
X_10_ Residential area 1 = urban (other is control group)	0.184***	0.087	1.20	− 0.108	0.132	0.90	0.292**	0.124	1.34
X_12_ Education 1 = Senior college and above	0.223***	0.124	1.25	0.197	0.159	1.22	0.438***	0.139	1.55
2 = Junior college	0.308***	0.101	1.36	0.035	0.142	1.04	0.18	0.133	1.20
3 = Senior high school (other is control group)	0.264***	0.096	1.30	− 0.026	0.146	0.97	0.186	0.138	1.20
Cox & Snell		0.419			0.430			0.424	
Nagelkerke		0.451			0.465			0.461	
McFadden		0.205			0.217			0.217	

Link function: Logit.

### Analysis of satisfaction probability

As indicated by the estimation results of Model (5) and Model (7) (See Tables 7, 8), in the case of food safety satisfaction, the probability of "average satisfaction" is greater than "relative satisfaction" and "relatively satisfaction". Horizontally (Model 7), the satisfaction of low-income residents with food safety has the highest probability of “quite satisfied”, followed by the residents with medium and high incomes residents. The variation trend of probability of “relatively satisfied” and “average satisfied” is consistent, and the high-income residents having the largest probability, followed by medium and low-income. That is to say, along with the rise of the income levels of residents with heterogeneous incomes, the probability of “quite satisfied” declines, and the probability of “relatively satisfied” and “average satisfied” rises. In terms of the vertical comparison of the estimation results of Model (7), the probability of “quite satisfied”, “relatively satisfied” and “average satisfied” for food safety satisfaction of heterogeneous residents increases gradually, which is consistent with the estimation results of Model (5).

### Analysis of the impacts of the basic information about investigated residents

In the estimation results of Model (5) and Model (7), the estimation results of gender, age, and occupation variables are negative values, and the OR values are less than 1. This indicates that the 3 variables exert significant negative impacts on dependent variables, the impacts of age and occupation are relatively complicated among them. In terms of the estimation results of Model (5), residents who are employed by enterprises and institutions and rural migrant workers in cities all passed the significance test. Residents whose occupations were technicians and service workers did not pass the significance test. Other variables passed the significance test, and the residents aged 16–30 had the most significant impacts, followed by residents whose occupation is rural migrant workers in cities. The significance of gender lies at the intermediate level. From the estimation results of Model (7), the impacts of residents aged 31–45 with heterogeneous incomes fail to pass the test, while other variables passed the significance test. In terms of income heterogeneity, male residents with medium and high incomes have a significant impact (-0.31), while residents with low incomes have a relatively insignificant impact (-0.168). This indicates that along with the change of gender, the probability of "relatively satisfied" in food safety satisfaction will decrease, and the gap between medium and high incomes is small. Why is that? On the one hand, under the influence of feudal traditional ideas, women are responsible for the management of daily basic life and have low expectations for the safety of food consumed by their families, which can be easily satisfied. On the other hand, influenced by traditional social labor division, men focus on social undertakings and shoulder the heavy burden of supporting the whole family, and have a high expectation of food safety. Low-income residents aged 16–30 have the most significant impacts, followed by the medium and high-income stratum. Residents aged 31–45 with heterogeneous incomes have insignificant impacts.

In Model (5) and Model (7), both the estimation results of educational background and residence place attributes are positive values, and the OR values are greater than 1. In the estimation results of Model (5), the impacts of senior college and above is the most significant (0.322), followed by junior college (0.213) and senior high school (0.201). The partial regression coefficient of residents of primary school and below has been set as 0 by the model. In terms of the estimation results of Model (7), the partial regression coefficients of residents with low incomes whose educational background is senior college and above, junior college, and senior high school are 0.223, 0.308, and 0.264 respectively. This is because there are a large number of residents with junior college diplomas in the low-income stratum; therefore, it has a significant impact on residents compared to other educational backgrounds. Educational background has an insignificant impact on residents with medium incomes, and they fail to pass the significance test. Among the high-income groups, college degree or above has a significant impact and passes the significance test. Other educational backgrounds have an insignificant impact and fail to pass the significance test. With the increase of income, the probability of "relatively satisfied" food safety satisfaction also increases for the residents with a college degree or above high-income residents with a high degree of education boast a rich knowledge reserve and a strong ability to identify average harmful food, therefore having a significant impact on food safety satisfaction. In other words, along with the rise of income level and educational background, the probability of “quite satisfied” food safety satisfaction rises correspondingly.

As indicated by the estimation results of the variable of residential place attribute in Model (5) and Model (7) (See Tables 7, 8), the attribute of settlements is that urban residents have a significant impact on food safety satisfaction., and the OR value is greater than 1. In terms of the estimation results of residents with heterogeneous incomes (Model 7), the partial regression co-efficient of urban low-income residents (0. 184) was less than that of high-income stratum (0.292), and the impact of medium incomes is insignificant and fails to pass the significance test. This indicates that the probability of "relatively satisfied" food safety satisfaction of urban high-income residents is more significant than low-income residents. According to survey findings, cities have a more mature system of food safety supervision than rural areas, and urban residents pay more attention to food safety than rural residents.

### Analysis of impacts of social environment

According to the estimation results of the social environment variable of Model (5) and Model (7), the partial regression coefficient of variables such as special correction and attention is negative, and the OR value is less than 1. In model (7), the estimated results of the middle-income class are not significant and cannot pass the significance test. The absolute value of the specially modified partial regression coefficient in model (5) is larger than that in model (7) and smaller than that in model (7). This indicates that the special rectification variable has a significant impact on the food safety satisfaction of residents with high incomes. If another unit is added to special rectification, the increase times of “quite satisfied” probability satisfaction will be greater than the change of low-income group. The attention variable has an insignificant impact on the food safety satisfaction of residents and failed to pass the significance test.

According to the estimation results of Model (7) (See Table 8), such variables as supervision, publicity, punishment intensity and complaint treatment and other variables have a significant positive impact on the satisfaction of food safety, and the impact is statistically significant, with OR value greater than 1. The impacts of the social environment factor on the food safety satisfaction of residents with heterogeneous incomes are quite complicated. In terms of vertical comparison (See Table 8), supervision has the most significant impact on the food safety satisfaction of residents with low incomes, followed by punishment intensity, complaint treatment, and publicity. The impact of each variable on high-income residents is consistent with the ranking of low-income groups, and publicity has the most significant impact among the middle-income group, followed by punishment intensity, supervision, and complaint treatment.

In a horizontal comparison, the supervision variable has the most significant impact on the high-income stratum, followed by the medium-income stratum, and the low-income stratum has the most insignificant impact. Publicity has the most significant impact on the low-income stratum, followed by the medium-income and high-income stratum. Punishment intensity has the most significant impact on the high-income stratum, followed by the low-income and medium-income stratum. The variable of complaint treatment has a more significant impact on medium-income stratum than low-income stratum but has an insignificant impact on high-income stratum. As a whole, with the improvement of residents’ income level, the supervision and punishment intensity have a more and more significant impact on the food safety satisfaction of residents with heterogeneous incomes. The impacts of publicity and complaint treatment on the food safety satisfaction of residents with heterogeneous incomes assume a change trend of “inverted V-shape”.

## Discussion

### Income heterogeneity and food safety satisfactions

From the estimation results of Model (7), it can be seen that China is facing a quite serious issue of food safety. Residents with heterogeneous income have the lowest probability of "relatively satisfied" and the highest probability of "average satisfied" in food safety satisfaction. The higher the income of residents with heterogeneous incomes is; the lower their food safety satisfaction would be. According to field survey findings, residents with medium and high incomes among residents with heterogeneous incomes have a higher income and can afford a richer variety of food than low-income stratum, so the probability of buying unsafe food also increases correspondingly, thus reducing the residents’ satisfaction with food safety. Next, residents with medium and high incomes have a relatively high degree of education and a stronger ability of discrimination to food safety. Even if they cannot discrimination by themselves, they have access to social resources that are more abundant than low-income groups to distinguish food safety; therefore, having a significant impact on food safety satisfaction. Relatively, under the influence of educational background and social environment, low-income groups have a weak ability to distinguish food safety. In other words, although they purchase unsafe food unwittingly, this did not exert a huge impact on their food safety satisfaction. On the other hand, low-income residents have a lower income and purchase food less frequently than the high-income stratum, so they less likely to be “dissatisfied” with food safety satisfaction. Besides, subject to the influence of traditional dietary consumption habits, the low-income stratum assumes a self-sufficiency feature in basic life food including vegetable, flour, and steamed buns, and with a sufficient mastery of production process information. This promotes the satisfaction of low-income residents with food safety. Zhang had a similar conclusion while studying the preference and satisfaction of rural public service demand at different income levels^20^. Due to the influence of characteristics such as educational background, living environment, income level and other features of residents with heterogeneous incomes, there are significant differences in food safety satisfaction of residents with heterogeneous income.

### Income difference and right-safeguarding consciousness

As indicated by statistical data, residents do not have a stronger consciousness of right-safeguarding in food consumption. The higher the income of residents with heterogeneous income, the stronger their awareness of rights protection will be. According to survey findings, the medium-level stratum shows the highest percentage of right-safeguarding behaviors (71.5%) in face of infringement, followed by the high-income stratum (69.2%) and low-income stratum (65.4%). To explore the cause of this phenomenon, the residents with heterogeneous incomes were interviewed. The results of the interview indicate that the low consciousness of food consumption right-safeguarding is mostly attributed to the low price of relevant food, and which does not bring serious harm to the health of consumers In general, residents with heterogeneous incomes will choose to abandon unsafe food, or solve the problem through negotiation with merchants.

Medium-income residents have the highest consciousness of right safeguarding. On the one hand, compared with low-income stratum, medium-income residents have a higher degree of education and tend to use the corresponding right-safeguarding way. On the other hand, compared with high-income stratum, medium-income residents pay a lower opportunity cost when they choose to safeguard their rights. High-income residents have a medium level of awareness of safeguarding their rights which mainly because high-income and medium-income residents have a higher education level, so they know which food safety issues are torts and how to safeguard their rights.

Next, high-income residents is not positive about the choice of low-value consumer goods as their high-income level. Besides, due to work reasons, high-income residents mostly choose to waive right-safeguarding in face of infringement which involves a huge opportunity cost. Low-income residents have the weakest right-safeguarding consciousness, and 34.6% of them do not take any right-safeguarding actions. On the one hand, low-income residents do not know how to safeguard their rights. On the other hand, low-income residents cannot to identify unsafe food. Therefore, residents with heterogeneous incomes show different behaviors in face of food safety infringements.

## Conclusion and suggestion

The results show that residents are generally worried about food safety, and there is a strong demand for safe food. Gender, age, educational background, supervision, publicity, and complaint handling significantly affect residents’ food safety satisfaction. In terms of income heterogeneity, low-income residents have the highest degree of satisfaction with food safety. With the improvement of income level, their degree of satisfaction is decreasing. Residents of high-income groups have a strong sense of safeguarding their rights after buying unsafe food. With the decline of income level, so does their awareness of safeguarding rights. In terms of the right-safeguard way, the majority of interviewees choose to return or refund money after consulting with the seller. There is no punishment or too little punishment for illegal sellers, which is one of the most important reasons that lead to frequent food safety in China.

Based on the conclusion of the research, the following policy suggestions are proposed:

- Set up an all-round system of food safety supervision system, which including such links as food production, circulation and marketing, at the same time, to control the origin channel of harmful food to flow to residents (consumers), promote food safety and quality, and to promote food safety satisfaction among the residents;
- According to the survey, more than 60% of workers in food processing enterprises are unclear about the quality standards of their work. Therefore, it is suggested that we should reinforce supervision of food production, processing, marketing, and other systems, and carry out a strict system of punishment management system for the technical training of workers, and standardize the food production, promote the safety and quality of food, and enhance the food safety satisfaction of residents;
- As indicated by the survey data, nearly 35% of residents fail to take any right-safeguarding action when suffering from infringement, which is also a connivance of illegal food safety laws to some extent. Nearly 40–50% of residents choose to directly negotiate with operators due to complaint cost, time, and other reasons, although it’s not easy to get the desired effect and physical conflicts tend to occur. It is suggested that we should set up interactive multi-media information pavilions to collect resident complaints, and issue a work schedule and treatment results for these complaints regularly, to shorten right-safeguarding formalities and reduce right-safeguarding cost, and to stimulate the right-safeguarding consciousness of residents with heterogeneous incomes, and encourage social forces to monitor and report food safety problems and promote the satisfaction of food safety.

Food security is not only a problem for China, but also a hot issue concerned by the whole world. In particular, the sudden outbreak of COVID-19 will further intensify the competition in the world food trade and attract the attention of governments all over the world. Our research results are based on the analysis of 8000 survey samples in Xinjiang. Although it cannot reflect the overall situation of food safety in China, to a certain extent, it can reflect the food consumption situation of residents in border minority areas. In future studies, we can select samples from the eastern, central, and western regions. By expanding the survey scope, we can reveal the food consumption status of the whole Chinese population. In terms of research perspective, in addition to the analysis from the perspective of residents’ income, the study can also be conducted from the perspective of the difference between rural and urban residents’ consumption behaviors. In addition, we can also analyze the change of residents’ food consumption behavior in the future by combining the influence of the continuous spread of COVID-19.
